# The Regulatory Network of Cyclic GMP-AMP Synthase-Stimulator of Interferon Genes Pathway in Viral Evasion

**DOI:** 10.3389/fmicb.2021.790714

**Published:** 2021-12-13

**Authors:** Tongyu Hu, Mingyu Pan, Yue Yin, Chen Wang, Ye Cui, Quanyi Wang

**Affiliations:** ^1^State Key Laboratory of Natural Medicines, Department of Life Science and Technology, China Pharmaceutical University, Nanjing, China; ^2^Division of Immunology, The Boston Children’s Hospital, Boston, MA, United States; ^3^Department of Pediatrics, Harvard Medical School, Boston, MA, United States

**Keywords:** viral evasion, cGAS-STING, type I interferon, innate immune, post-translational modification

## Abstract

Virus infection has been consistently threatening public health. The cyclic GMP-AMP synthase (cGAS)-Stimulator of Interferon Genes (STING) pathway is a critical defender to sense various pathogens and trigger innate immunity of mammalian cells. cGAS recognizes the pathogenic DNA in the cytosol and then synthesizes 2′3′-cyclic GMP-AMP (2′3′cGAMP). As the second messenger, cGAMP activates STING and induces the following cascade to produce type I interferon (IFN-I) to protect against infections. However, viruses have evolved numerous strategies to hinder the cGAS-STING signal transduction, promoting their immune evasion. Here we outline the current status of the viral evasion mechanism underlying the regulation of the cGAS-STING pathway, focusing on how post-transcriptional modifications, viral proteins, and non-coding RNAs involve innate immunity during viral infection, attempting to inspire new targets discovery and uncover potential clinical antiviral treatments.

## Introduction

Infectious diseases are the top threats to public health. With the persistent invading of various pathogens, mammalians have developed certain strategies to protect themselves from extraneous risks. During infection, germline-encoded pattern recognition receptors (PRRs) recognize the pathogen and damage-associated molecular patterns (PAMPs and DAMPs), such as the viral nucleic acids (DNA or RNA), which initiate subsequent immune responses, and orchestrate an elaborate defense system against infection (Carty et al., 2021; Li and Chang, 2021).

The cGAS-STING pathway is one of the predominant and conserved mechanisms of the host to eliminate pathogens through several aspects, including IFN-I, releasing, autophagy initiation, pro-inflammatory cytokines production, and cell death pathways (Phelan et al., 2020; Fang and Peng, 2021; Zhao et al., 2021). Viruses have optimized their evading tactics for superior replication and spreading to counteract host immunity. For instance, due to their genetic flexibility, viruses have developed various viral proteins and non-coding RNAs to interrupt several checkpoints of cGAS-STING. Besides, they craftily confuse the host regulatory system to diminish immune responses and engage viral escaping from immunity (Li et al., 2019; Bouayad, 2020; Kikkert, 2020).

The models of most immune evasion during innate immune responses are conserved, including altering the post-transcriptional modifications (PTMs) of vital proteins to inactivate or degrade these components, eluding DNA sensing from PRRs, decreasing cyclic GMP-AMP (cGAMP) cellular abundance, and modifying metabolism approach in host cells (Eaglesham and Kranzusch, 2020). Intriguingly, novel mechanisms are continuously uncovered, which elucidate a more concrete picture of cGAS-STING involvement in viral evasion. Nonetheless, how pathogens avoid detection and clearance by immune systems needs to be more comprehensively elucidated. Herein, we have summarized newly emerging hot spots of cGAS-STING regulation in viral evasion and summarized frontier advances in relevance processes. The present review provides potential worth evaluating targets in innate immune response that are viable in clinical trials and antiviral reagents development for current and future studies.

## The Cascade of Cyclic GMP-AMP Synthase-Stimulator of Interferon Genes Pathway During Viral Invasion

**FIGURE 1:**
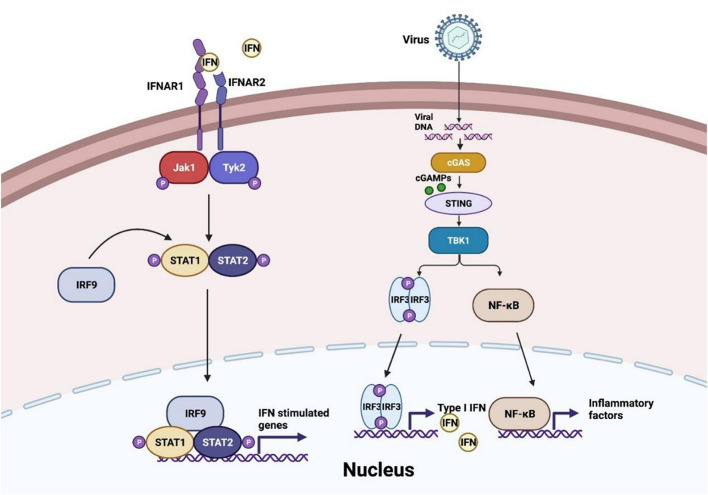
cGAS-STING pathway in virus invasion. After virus infection, cyclic GMP-AMP synthase (cGAS) senses the viral DNA to synthesize cyclic GMP-AMPs (cGMPs) and activate stimulator of interferon genes (STING) to recruit TANK-binding kinase 1 (TBK1) and IκB kinase (IKK), and then interferon regulatory factor 3 (IRF3) and nuclear factor-κB (NF-κB) are induced to translocate into the nucleus and generate the production of IFN-I-β and inflammatory factors. IFN-I-β conducts Janus kinase signal transducer and activator of transcription (JAK-STAT) pathway activation and initiates interferon-stimulated genes transcription.

At the first stage of viral invasion, virus-derived double-stranded DNAs (dsDNAs) trigger the conformational change and activation of cGAS. Activated cGAS catalyzes and releases the second messenger 2′,3′-cGAMP, which binds to STING, an adaptor located at endoplasmic-reticulum (ER)-membrane (Chen et al., 2016). This process induces oligomerization of STING and its traveling from ER to the Golgi via ER-Golgi intermediate compartment (ERGIC) (Hopfner and Hornung, 2020). Translocation and structure switch of STING provides the prerequisite for TANK-binding kinase 1 (TBK1) recruitment and auto-phosphorylation. TBK1 induces phosphorylation of STING C-terminal tail (CTTs) motif, which supplies a docking site for interferon regulatory factor 3 (IRF3) (Shang et al., 2019; Zhao et al., 2019). Moreover, recent studies suggest that recruitment of TBK1 to STING may perform a more significant role in antagonist virus infection and restrict oncogenesis (Figure 1), which expands the horizon of cGAS-STING axis function besides IRF3 and nuclear factor-κB (NF-κB) signaling (Yum et al., 2021). Accordingly, IRF3 is phosphorylated by TBK1 and subsequently dimerized, resulting in IRF3 nuclear translocation and transcriptional activation, further inducing IFN-I release (Zhang et al., 2020c). Besides, STING activation also contributes to the recruitment of IκB kinase (IKK) and facilitates NF-κB inhibitor IκBα phosphorylation. Activated NF-κB, similar to activated IRF3 dimer, translocates into the nucleus and generates downstream pro-inflammatory cytokines formation (Motwani et al., 2019).

As a signaling cascade of pathogen sensing and (IFN-I) production, IFN-β binds to IFNα/β receptor 1 (IFNAR1) and IFNAR2, which launch interferon-stimulated genes (ISGs) production through Janus kinase signal transducer and activator of transcription (JAK-STAT) pathway (Wan et al., 2020). Activated JAK1 and tyrosine kinase 2 (TYK2) trigger signal transducers and activators of transcription 1 (STAT1)/STAT2 phosphorylation. IRF9 is then recruited to STAT1/2 heterodimers to constitute the IFN-I-stimulated gene factor 3 (ISGF3) complex and initiate ISGs production (Schneider et al., 2014). In conclusion, the autocrine and paracrine of IFN-I-β consist of dynamic feedback to regulate the cGAS-STING axis (Wang et al., 2020a).

Additionally, cGAS-STING signaling participates in IRF3-independent viral-sensing and triggers autophagy, an ancestral antiviral process of host defense (Yamashiro et al., 2019; Hopfner and Hornung, 2020). The interaction of STING and LC3 leads to non-canonical autophagy initiation without classic autophagy regulators, such as Unc-51-like kinase 1 (ULK1) protein kinase complex (Birgisdottir et al., 2013; Liu et al., 2019). Golgi-oriented STING vesicle trafficking provides a conjugation and lipidation membrane platform for LC3, enabling autophagosome formation (Gui et al., 2019). Besides, STING activation also results in V-ATPase-ATG16L1-induced LC3B lipidation on single-membrane perinuclear vesicles, presumably to cripple invaded viruses by facilitating lysosomal fusion (Fischer et al., 2020). Furthermore, the recent advance of crosstalk between STING and autophagy indicates that STING translocating to the Golgi is indispensable to switch on DNA- and RNA-virus triggered autophagy (Zhang R. et al., 2021). In addition, cGAS is demonstrated to interact with the autophagy protein Beclin-1 as well. This event suppresses the cGAMP synthesis and releases the RUBICON, an autophagy inhibitor, which increases autophagic clearance of viral DNA to prevent the overactivation of cGAS-STING signaling (Liang et al., 2014).

Accumulating studies show that the cGAS-STING pathway is widely implicated in the DNA-sensing process, including viral clearance and autoimmune diseases (Lin and Zheng, 2019; Brezgin et al., 2021; Zheng, 2021). It is important to broaden our comprehension of each step of the cGAS-STING signaling to finetune the immune responses. Moreover, utilizing this pathogen defense pathway supplies valuable guidance to predict potential antiviral therapeutics and drug developments, which hinders viral evasion from host innate immune surveillance.

## Post-Translational Modification of Cyclic GMP-AMP Synthase-Stimulator of Interferon Genes Pathway Components Is Significant to Avoid Viral Evasion

Several studies showed that viruses benefit from hijacking host enzymes to alter the modification of antiviral immune cascades component proteins, thus conducive to their replication (Carty et al., 2021; Hong et al., 2021; Song et al., 2021). Understanding the details of PTMs in cGAS-STING signaling helps interrupt virus evasion. Here we summarize the current findings of PTM regulating on different components in the cGAS-STING pathway.

### Post-transcriptional Modifications of Cyclic GMP-AMP Synthase Ensure Functional Protection From Viral Infection

When the viral DNAs are released in the cytoplasm, cGAS efficiently recognizes and processes the enthetic DNA ligands to synthesize cGAMP. Virus infection has been found to induce various forms of post-translational modifications at different sites of cGAS, which manipulates the synthetase capability of cGAS and its affinity to pathogenic DNA (Table 1; Wu and Li, 2020; Bhowmik and Zhu, 2021; Song et al., 2021).

**TABLE 1:** Enzymes involved in cGAS PTMs.

Target protein	PTM sites (h:human, m:mice)	PTM	Enzymes	Function	References
cGAS	T68, S213(h)	Phosphorylation	DNA-PK	Hinders cGAS oligomerization	Sun et al., 2020
	E272(m)	Polyglutamylation	TTLL6	Impedes cGAS DNA-binding ability	Xia et al., 2016
	E302(m)	Mono-glutamylation	TTLL4	Suppresses cGAS synthase activity	Xia et al., 2016
	E272(m)	Depolyglutamylation	CCP6	Activates cGAS	Xia et al., 2016
	E302(m)	Demonoglutamylation	CCP5	Activates cGAS	Xia et al., 2016
	K335/372/382(m)	De-SUMOylation	SENP7	Reverses cGAS inhibition	Cui et al., 2017
	R124(m)	Methylation	PRMT5	Interrupts cGAS binding with DNA	Ma et al., 2021
	K384/394/414(h)	Deacetylation	HDAC3	Maintains cGAS response to DNA	Dai et al., 2019
	K47/52/62/83(m)	Acetylation	KAT5	Increases DNA-binding of cGAS	Song et al., 2020
	K231/421(h)	Polyneddylation	RNF111	Improves cGAS dimerization	Li et al., 2021b

To maintain the homeostasis of cGAS, several PTMs play an important role in down-regulating cGAS function to prevent its excessive activation. Upon virus infection, cGAS is phosphorylated by DNA-dependent protein kinase (DNA-PK), hindering its oligomerization and enzymatic activity (Sun et al., 2020). The glutamylation of cGAS is also identified after DNA virus infections. The dynamic regulation of cGAS glutamylation, either mono-, or poly-, impedes its synthase activity and DNA binding capability, which adjusts the strength of immune response to pathogens (Xia et al., 2016). The enzymes involved in cGAS glutamylation are concluded in Table 1. Additionally, recent research revealed cGAS was symmetrically dimethylated at Arg124 residue by protein arginine methyltransferase 5 (PRMT5). As a result, cGAS could not bind the DNA ligands, and the antiviral response was dampened during HSV-1 infection (Ma et al., 2021).

Proper activation of cGAS is important to generate IFN-I production and subsequent antiviral immunity. Sentrin/SUMO-specific protease 7 (SENP7) rescues cGAS inhibition by removing the small ubiquitin-like modifier (SUMO) from Lys335, 372, and 382 of cGAS (Cui et al., 2017). The activity of cGAS can also be dynamically regulated by acetylation. Acetylation of cGAS at Lys384, Lys394, and Lys414, which are close to its C terminal, keeps cGAS at a quiescent state. Sensing abnormal DNA ligands triggers histone deacetylase 3 (HDAC3) to deprive the acetyl groups of cGAS to switch on its enzymatic activity (Dai et al., 2019). In contrast, lysine acetyltransferase 5 (KAT5) mediating the acetylation on the N terminal of cGAS at Lys47/52/62/83 promotes the activation of cGAS (Song et al., 2020). Contemporary research has exhibited the poly-neddylation of cGAS with the presence of the Ube2m-Rnf111 axis. The neddylation of cGAS conserved residues K231 and K421 are crucial to facilitate cGAS dimerization and promote its cytoplasmic DNA binding ability. As a result, these modifications restrict HSV-1 infection *in vivo* (Li et al., 2021b).

Acting as the initial DNA-sensor to induce robust innate immune responses, the enzymatic activity and stability of cGAS are precisely regulated. To protect the host from the threat of virus, more PTM forms and sites of cGAS need further investigation.

### The Modification of Stimulator of Interferon Genes by Ubiquitin Maintains Stimulator of Interferon Genes Relevant Antiviral Responses

STING, the adaptor downstream of cGAS, is also the center molecule of most DNA-sensing pathways. Multiple PTMs have been found to dominate STING intracellular trafficking, conformational change, and activation during infection (Li et al., 2020; Hong et al., 2021). One of the most common modifications that dynamically regulates STING activity is ubiquitination. Different STING ubiquitin linkage types accomplish diverse functions to STING relevance immune cascades (Davis and Gack, 2015). In this section, we focus on the ubiquitination of STING during viral infection (Table 2).

**TABLE 2:** Dynamic ubiquitin decorations of STING.

Target protein	PTM sites	Enzyme	Decoration type	Function	References
STING	K288/337	TRIM29	Ubiquitination (K48)	Promotes STING proteasomal degradation	Li et al., 2018
	K150	RNF90	Ubiquitination (K48)	Induces STING degradation	Yang et al., 2020
	K236	USP44	Deubiquitination (K48)	Promotes STING stability	Zhang et al., 2020a
	K347	OTUD5	Deubiquitination (K48)	Prevents STING degradation	Guo Y. et al., 2021
	K20/150/224/236	TRIM32	Ubiquitination (K63)	Increases STING interaction with TBK1	Zhang et al., 2012
	–	USP49	Deubiquitination (K63)	Improves STING aggregation and translocation	Ye et al., 2019
	K150	MYSM1	Deubiquitination (K63)	Attenuates STING activation	Tian et al., 2020

STING can be ubiquitinated with K48-linked polyubiquitination, which leads to its proteasome degradation. The immune system has evolved this strategy as a negative feedback loop to balance normal immune responses and autoimmunity. TRIM29, an E3 ligase, can be induced by DNA virus stimulation, which catalyzes K48-linked ubiquitination of STING on Lys288/337 and mediates STING degradation (Li et al., 2018). Similarly, K48-linked ubiquitination of STING at Lys150 by RNF90 also negatively regulates the DNA-sensing pathway (Yang et al., 2020). The ubiquitination mediated degradation process can be reversed by deubiquitinases, which maintain STING function. The deubiquitinases OTUD5 and ubiquitin-specific protease (USP) 44 deprive the K48-linked polyubiquitin chains of STING at Lys347 and Lys236, respectively (Zhang et al., 2020a).

Distinct from K48 ubiquitination mediated degradation, K63-linked ubiquitination promotes the activation of the substrates. STING can also be modified by K63-linked ubiquitination at Lys20/150/224/236 by tripartite motif protein 32 (TRIM32), which of these are essential for STING activation and interaction with TBK1 (Zhang et al., 2012). On the contrary, USP49 antagonizes STING activation by removing its K63-linked ubiquitin chains, impedes STING aggregation, and subsequent TBK1 recruitment after HSV-1 invasion (Ye et al., 2019). Similarly, infected by DNA virus, the Myb-like, SWIRM, and MPN domains 1 protein (MYSM1) is increasingly expressed and interacts with STING, leading to the removal of K63-linked ubiquitination STING at Lys150 to down-regulate STING signaling (Tian et al., 2020). Intriguingly, a recent observation reveals that a novel autophagy receptor, CCDC50, can recognize K63-polyubiquitinated STING for autophagic degradation, which inhibits IFN-I and pro-inflammatory cytokines production. Moreover, CCDC50 deficiency restricts HSV-1 replication, which shows a possible therapeutic strategy to prevent viral evasion (Hou et al., 2021).

STING is standing at the crossroad of IFN-I releasing, non-classical autophagy initiation, and NF-κB activation. The ubiquitin-related regulation is quite crucial for the stability and function of STING to prevent viral immune evasion.

### Appropriate Modification of TANK-Binding Kinase 1 Defends Viral Invasion

TBK1, the downstream component of STING in the cGAS-STING axis, its kinase activity is indispensable to IFN-I generation and virus-induced autophagy initiation (Sparrer et al., 2017). Multi-categories of post-translational modifications of TBK1 involve modulating the strength of cGAS-STING signaling activation (Table 3).

**TABLE 3:** Modifications of TBK1 in regulating IFN-I production.

Target protein	PTM sites	PTM	Enzymes	Function	References
TBK1	K30/401	Ubiquitination (K63)	RNF128	Activates TBK1	Song et al., 2016
	K670	Deubiquitination (K33)	USP38	Induces subsequence ubiquitination	Lin et al., 2016
	K670	Ubiquitination (K48)	DTX4 and TRIP	Promotes TBK1 degradation	Lin et al., 2016
	K30/401	Deubiquitination (K63)	USP15	Represses TBK1 activation	Huang et al., 2020
	K344	Ubiquitination (K27)	NEDD4	Induces selective autophagy of TBK1	Xie et al., 2021
	K241/692	Deacetylation	HDAC3	Enables TBK1 kinase activity	Tang et al., 2021
	C637	S-glutathionylation	GSTM1	Inhibits TBK1 phosphorylation	Wang et al., 2020b
	W354/394	Tyrosine-phosphorylation	Lck/Hck/Fgr	Impedes TBK1 activation	Liu et al., 2017

Ubiquitination and acetylation are also engaged in TBK1 regulation. Virus sensing induced the expression of E3 ubiquitin ligase RNF128. K63-linked ubiquitin chains are continuously added to TBK1 sites at Lys30 and Lys401, triggering TBK1 activation and following IFN-I-β release (Song et al., 2016). USP38 exclusively removes K33-linked poly-ubiquitination of TBK1 at Lys670, which is consecutively replaced by K48-linked ubiquitin chains attributed by DTX4 and TRIP, causing the proteasomal degradation of TBK1 (Lin et al., 2016). Additionally, with the assistant of UBE2S, USP15 is recruited to TBK1 and removes K63-linked polyubiquitin chains of TBK1. This process represses IFN-I-β production and provides an advantageous element for virus proliferation (Huang et al., 2020). Another reported E3 ubiquitin ligase, ASB8, is a negative regulator of IFN-I signal transduction. Mechanistically, after viral infection, ASB8 interacts with TBK1/IKKi kinase complex and promotes the K48-linked ubiquitination of TBK1/IKKi, which is degraded by proteasome afterward (Guo et al., 2020). Furthermore, NEDD4 drives the K27-linked poly-ubiquitination of TBK1 at Lys344 to instigate selective autophagy clearance of TBK1 (Xie et al., 2021). HDAC3 also involves deacetylation of TBK1 at Lys241 and Lys692, enabling TBK1 kinase activity (Dai et al., 2019). In turn, TBK1 mediates HDAC3 phosphorylation to enhance the deacetylase activity of HDAC3, which generates a feedback mechanism. The deficiency of HDAC3 impairs IFN-I releasing, therefore promoting viral replication in mice (Tang et al., 2021).

Several enzymes engage in distinct TBK1 modifications to prevent TBK1 from excessive activation upon viral stimulation. Previous studies reveal that the Src family kinases (SFKs) Lck, Hck, and Fgr restrict IFN-I production. During virus infection, Lck/Hck/Fgr can directly phosphorylate TBK1 at Tyr354/394 to restrain TBK1 dimerization and activation as a feedback approach in antiviral immunity (Liu et al., 2017). Recently, Wang et al. (2020b) investigated that a highly conserved cysteine residue C637 of TBK1 could be S-glutathionylated by glutathione S-transferase M1 (GSTM1). This special modification of TBK1 inhibits its phosphorylation at Ser172, hence regulating the release of IFN-I in the process of virus infection (Wang et al., 2020b).

### The Non-canonical Post-translational Changes of Interferon Regulatory Factor 3 Affect Immune Evasion of Virus

Last but not the least component regulated by PTMs in the cGAS-STING pathway is IRF3. The transcription factor IRF3 plays a commander-like role in manipulating *IFN-*β transcription upon viral infection. After translocated into the nucleus, IRF3 interacts with CREB-binding protein (CBP)/p300 to initiate downstream genes transcription (Schwanke et al., 2020). During pathogens infection, the host facilitates PTMs to alter the conformation and activity of IRF3, which accordingly performs antagonism between viruses and the host immune signaling (Table 4).

**TABLE 4:** Modifications of IRF3 during virus infection.

Target protein	PTM sites (h:human,m:mice)	PTM	Enzymes	Function	References
IRF3	K98 (h)	Deubiquitination (K6)	OTUD1	Restricts IRF3 DNA binding	Zhang et al., 2020e
	K193/360/366(m)	ISGylation	HERC5	Stabilizes IRF3	Shi et al., 2010
	K366(m)	Monomethylation	NSD3	Ensures IRF3 subsequent phosphorylation	Wang et al., 2017
	K366(m)	Dephosphorylation	PP1cc	Dephosphorylate IRF3	Wang et al., 2017
	K359(m)	Acetylation	KAT8	Reduces IRF3 induced gene transcriptions	Huai et al., 2019

Besides phosphorylation and classical ubiquitination, increasingly novel PTMs are verified of their involvements in regulating IRF3 intention. Atypical ubiquitination in cGAS-STING signal cascades is still barely reported. Zhang et al. (2020e) identified the K6-linked ubiquitination of IRF3 at Lys39/98/105 under viral infection, which is essential for its DNA binding ability. Moreover, Zhang et al. (2020e) team uncovered that the ovarian tumor domain-containing 1 (OTUD1) can deubiquitinate K6-, K11-, and K29-linked ubiquitination of IRF3. The ubiquitin-like protein ISG15 conjugates with target proteins and induces ISGylation, proven crucial during viral invasion and evasion (Dzimianski et al., 2019; Chiang et al., 2021; Mathieu et al., 2021). The ISG15 E3 ligase HERC5 adds ISG15 to IRF3 at Lys193/360/366 to counteract with Pin1 induced IRF3 polyubiquitination, guaranteeing the stability of an IRF3 structure (Shi et al., 2010). Nuclear receptor-binding SET domain 3 (NSD3) directs the Lys366 monomethylation of IRF3, shielding the phosphatase PP1cc-mediate IRF3 dephosphorylation, thus intensifying the transcriptional regulator function of IRF3 and following IFN-I release (Wang et al., 2017). IRF3 is also acetylated at Lys359 by lysine acetyltransferase 8 (KAT8) to attenuate virus-induced IFN-I generation. IRF3 acetylation interrupts its association with interferon genes promoters, hence invalidating over-committed immune response *in vivo* (Huai et al., 2019).

Collectively, accumulating evidence indicates that the post-translational modifications involved in the cGAS-STING pathway are quite significant. The improvement of protein structure analysis techniques has broadened the approaches for researchers to validate additional decorations of target proteins. A growing number of new PTMs and the relative functional aspects can be recognized. Delineating the complicated network of PTMs control of cGAS-STING can contribute to the present state of antagonizing viral evasion strategies investigations.

## Manipulation of Cyclic GMP-AMP Synthase-Stimulator of Interferon Genes Axis by Viral Proteins

**FIGURE 2:**
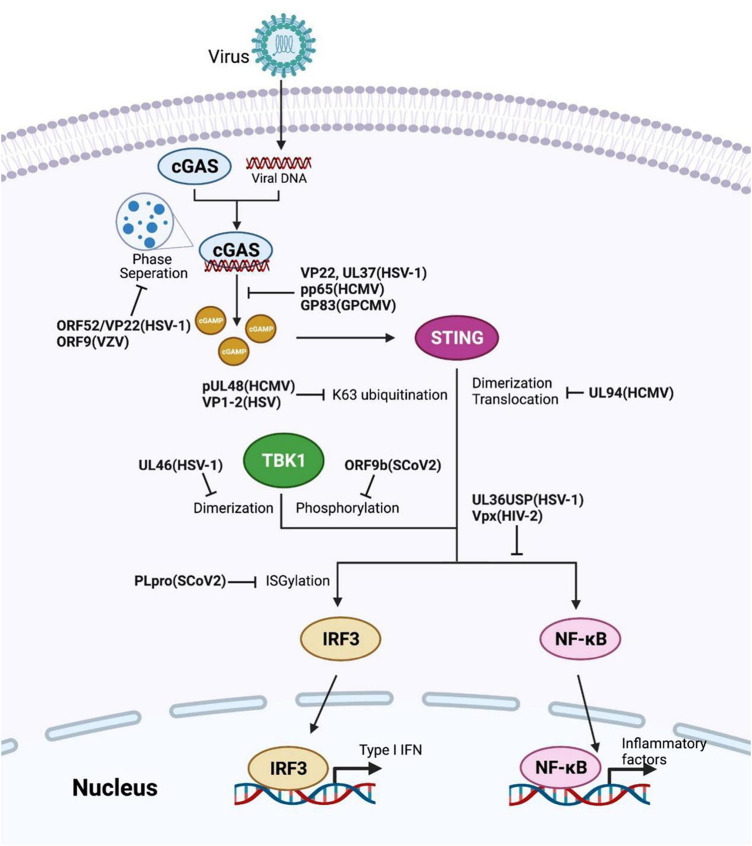
Viral proteins induced evasion strategies by restricting the cGAS-STING axis. Several reported viral proteins dysregulate different checkpoints in the cGAS-STING pathway, such as invalidating the enzyme activity of cGAS or TBK1, obstructing STING activation, altering the modifications of key components. The corresponding viral proteins and their species are labeled in bold.

Struggling with the defense mechanism of host immunity, viruses themselves have developed several means to create a more convenient environment for replication (Zhang et al., 2016; Lange et al., 2021; Sausen et al., 2021). For instance, viruses utilize their proteins to control the host’s innate-immune signaling pathways for evasion (Table 5). This section concentrates on the direct interaction between viral proteins and the pivotal component in the cGAS-STING pathway (Figure 2). Addressing critical interplay between virus and host interferon responses will contribute to further therapeutic procedure researches.

**TABLE 5:** Viral proteins involved in cGAS-STING cascade signaling.

Genre	Genome	Virus	Viral protein	Target proteins	Function	References
Deubiquitinase (DUB)	DNA	HCMV	pUL48	STING	Deubiquitination	Kumari et al., 2017
		HSV-1	VP1-2	STING		Bodda et al., 2020
			UL36USP	IκBα		Ye et al., 2017
	RNA	TGEV	PL1	STING		Hu et al., 2017
		SARS-CoV	PLpro	STING-TRAF3-TBK1 complex		Chen et al., 2014
		SARS-CoV2	PLpro	IRF3	De-ISGylation	Shin et al., 2020
Tegument protein	DNA	VZV	ORF9	cGAS	Restricts cGAS-DNA condensates	Xu G. et al., 2021
		HSV-1	UL37	cGAS	Impairs cGAMP synthesis	Zhang et al., 2018
			UL46	TBK1	Inhibits TBK1 dimerization	You et al., 2019
			UL41	cGAS	Degrades cGAS mRNA	Su and Zheng, 2017
			VP22	cGAS	Restrains cGAS catalyze activity	Huang et al., 2018
			ORF52/VP22	cGAS	Inhibits cGAS-DNA phase separation	Xu G. et al., 2021
		HCMV	UL23	STAT1	Impedes STAT1 phosphorylation	Feng et al., 2021
			UL94	STING	Inhibits STING dimerization	Zou et al., 2020
			pp65	cGAS	Hampers cGAMP synthesis	Biolatti et al., 2018
		GPCMV	GP83	cGAS	Inhibits cGAS activity	Choi et al., 2021
Accessory protein	DNA	HSV-1	Us11	Hsp90	Restricts Hsp90-TBK1 complex formation	Liu et al., 2018
	RNA	SCoV2	ORF3a	STING	Obstructs STING triggered NF-κB activation	Rui et al., 2021
			ORF9b	TBK1	Decreases TBK1 phosphorylation	Han et al., 2021
		HIV-2, SIV	Vpx	STING	Diminishes STNG function in NF-κB initiation	Su et al., 2019
		HIV-1	Vpr	Karyopherins	Dampens IRF3 and NF-kB nuclear translocation	Khan et al., 2020

### Viral Deubiquitinases in Regulating Cyclic GMP-AMP Synthase-Stimulator of Interferon Genes Pathway

The dynamic modification of host proteins maintains the homeostasis in innate immune signaling regulation. Multiple viruses encode viral deubiquitinase (DUB) to interrupt the antiviral responses in host cells, aiming to attenuate innate immune system activation, such as the cGAS-STING axis (Kumari and Kumar, 2018; Proulx et al., 2021).

pUL48, a DUB encoded by human cytomegalovirus (HCMV), removes K63 ubiquitination of STING to attenuate IFN-I induction and promote carcinogenesis (Kumari et al., 2017). Transmissible gastroenteritis virus (TGEV) also utilizes its papain-like protease (PL1) to antagonize IFN-β expression through deubiquitination of STING (Hu et al., 2017). Analogously, the herpes simplex virus (HSV) DUB VP1-2 immediately interacts with Lys150-ubiquitinated STING and removes its K63-linked ubiquitination, inducing viral evasion in the brain (Bodda et al., 2020). In addition, the HSV-1 ubiquitin-specific protease (UL36USP) dampens IκBα degradation via its deubiquitinase activity, further restricting NF-κB signaling activation to dysregulate host immune response. Likewise, UL36USP also decreases the IFN-β cascade response upon HSV-1 infection (Ye et al., 2017).

The severe acute respiratory syndrome coronavirus (Sars-CoV) membrane-anchored PLpro domain (PLpro-TM) is elucidated its function of disrupting STING-TRAF3-TBK1 complex formation, decreasing the ubiquitination level of the complex components, ultimately impairing IRF3 phosphorylation and dimerization (Chen et al., 2014). The papain-like protease (PLpro) domain of SCoV cleaves the viral polyprotein and promotes viral spreading (Klemm et al., 2020). Although sharing high sequence identity with SCoV-PLpro, PLpro of SCoV2 prefers to remove the ubiquitin-like protein ISG15 modification of the host protein rather than ubiquitin, reflecting a different tendency for substrate selection. Upon invasion, SCoV2-PLpro was implicated in IRF3 de-ISGylation to inhibit IFN-I production (Shin et al., 2020).

### The Viral Tegument Proteins in Suppressing Innate Immune Responses

The tegument is a protein cluster that fills the space between the nucleocapsid and the envelope expressed in all herpes viruses. The tegument proteins are essential for the viral envelope and viral DNA containing capsid transport (Yang et al., 2019). Apart from virus enveloping, different kinds of tegument proteins have evolved diverse strategies to suppress host immunity.

Lately, the tegument proteins of HSV-1 have been reported to be involved in disrupting cGAS-STING induced IFN-I production. For instance, UL37 deamidates a crucial asparagine site of cGAS to eliminate cGAMP composition, deactivating IFN-I initiation cascades after infection (Zhang et al., 2018). Another tegument protein, UL46 encoded by HSV-1, obstructs TBK1 dimerization, facilitating declining IFN-I production and leading to HSV-1 immune evasion (You et al., 2019). Moreover, during HSV-1 infection, the tegument protein UL41 acts as the RNase to degrade cGAS mRNA, which contributes to HSV-1 evading of DNA-sensing pathway (Su and Zheng, 2017). Phase separation is a result of forming large biomolecules clusters and lately has explicated its role in intracellular immune signal regulation (Xiao et al., 2021). VP22 of HSV-1 has previously determined its function in impeding the cGAMP synthetase activity of cGAS (Huang et al., 2018). Xu G. et al. (2021) further clarified that gamma- and alpha-herpes tegument proteins ORF52, VP22, and ORF9 effectively disrupt the cGAS-DNA condensation as cGAS-DNA phase separation inhibitors to countermeasure viral clearance in host cells.

The HCMV tegument protein UL23 interacts with STAT1 and hinders STAT1 phosphorylation from optimizing subsequent viral dissemination (Feng et al., 2021). UL94 of HCMV represses translocation and dimerization of STING to facilitate virus replication; UL82 and UL35 also antagonize cGAS-STING signaling separately at STING trafficking and TBK1 level (Fu et al., 2017; Fabits et al., 2020; Zou et al., 2020). Moreover, the HCMV pp65 (pUL83) selectively associates with cGAS and disrupts its following signal transduction with STING, supporting HCMV evading from innate immunity (Biolatti et al., 2018). In other species, Choi et al. (2021) uncovered the role of guinea pig cytomegalovirus (GPCMV) tegument protein GP83, the supposed HCMV pp65 homolog, in epithelial cell infection. GP83 interacts with the DNA sensors IFI16 and cGAS, particularly in targeting cGAS, which shares a conserved function with pp65 (Choi et al., 2021).

### Accessory Proteins of Viruses Against Host Antiviral Immunity

Accessory proteins have different purposes and quantities in many viruses and focus more on viral spreading, evasion, and host immune regulation rather than manipulating viral replication (Fang P. et al., 2021).

Upon infection, herpes simplex virus 1 (HSV-1) accessory protein Us11 prevents Hsp90 interaction with TBK1, disrupting Hsp90-TBK1 complex formation. Moreover, Us11 also induces TBK1 proteasomal degradation. These processes of Us11 facilitate HSV-1 replication by reducing IFN-I-β generation (Liu et al., 2018). Vpx, a virion-associated accessory protein encoded by human immunodeficiency virus-2 (HIV-2) and certain simian immunodeficiency virus (SIV), binds STING to facilitate NF-κB complex organization and inhibit NF-κB signal transduction (Su et al., 2019). Further investigation reveals that HIV-1 accessory protein Vpr manipulates innate immunity to promote HIV-1 replication, showing another virus’s evasion strategy. Mechanically, Vpr prevents IRF3 and NF-κB import to nuclear by interacting with karyopherin, hence antagonizing downstream antiviral responses (Khan et al., 2020). SARS-CoV-2 accessory protein ORF3a interacts with STING to diminish intranuclear p65 accumulation, impeding NF-κB signaling initiation without affecting IRF3 triggered IFN-I generation (Rui et al., 2021). Han et al. (2021) reported that SARS-CoV-2 ORF9b interacted with TBK1 and dysregulated TBK1 phosphorylation to evoke escaping SARS-CoV-2.

Diverse viral proteins are a virus-involved strategy against host immunity. The persistent appearance of novel variants of viruses causes health emergencies to humanity. Mastering the function of viral proteins and how they manipulate the innate immune system helps us prevent viral evasion and ultimately reduce risks from virus-induced infection.

## Regulation of Non-Coding RNA in Viral Evasion

RNA-centric management of host-virus interactions is increasingly causing attention (Gokhale et al., 2021). This section has a brief systematical review of the function and mechanism of non-coding RNA implicated in virus-induced immune defense underlying the cGAS–STING pathway (Table 6).

**TABLE 6:** Regulations of non-coding RNA in virus invasion.

Type	Species	ncRNA	Function	References
lncRNA	Human and mouse	lncRNA-GM	Represses TBK1 S-glutathionylation	Wang et al., 2020b
circRNA	Human	AIVR	Increases CREBBP expression	Qu et al., 2021
miRNA	Teleost fish	miR-15b	Represses TBK1 expression	Chang et al., 2020
	Teleost fish	miR-210	Represses STING expression	Xu et al., 2018
	Human	miR-576-3p	Decreases STING expression	Geddes et al., 2018
	Cat	miR-101, miR-26a	Downregulate SOCS5	Zhang et al., 2020b
	HCMV	miR-US33as-5p	Disrupts IFN-IAR1 function	Zhang Q. et al., 2021
	HSV-1	miR-H2-3p	Suppresses DNA sensing by DDX41	Duan et al., 2019

Host immunity engages diverse RNA-directive strategies to operate vital proteins expression in IFN-I signaling. For instance, long non-coding RNAs (lncRNAs) contain more than 200 nucleotides and regulate gene expression at transcriptional or post-transcriptional levels (Kesheh et al., 2021). After being infected by Kaposi’s sarcoma-associated herpes virus (KSHV), the lncRNA NEAT1 and HEXIM1 from a special ribonucleoprotein complex interact with cGAS is required to initiate foreign DNA triggered cGAS-STING activation. The viral protein ORF52 can disrupt the interplay of HEXIM1-cGAS and induce KSHV evasion of immune response (Morchikh et al., 2017). Using functional screening of host lncRNAs, Wang et al. (2020b) revealed lncRNA-GM, an enhancer of TBK1 activity by interacting with glutathione S-transferase M1 (GSTM1), turns to reduce TBK1 S-glutathionylation. Virus invasion represses the abundance of lncRNA-GM in host macrophages, facilitating immune escaping under viral infection (Wang et al., 2020b). Moreover, Qu et al. (2021) reported that the circular RNA (circRNA) AIVR, an innovative lncRNA expressed in A549 cells, absorbed the microRNA (miRNA) bound to the positive regulatory protein of IFN-I-β generation, CREBBP. Deficient in AIVR expression dampens antiviral reaction in host cells (Qu et al., 2021).

The miRNAs are members of small non-coding RNAs. Multiple DNA and RNA viruses are capable of encoding miRNA to accelerate their propagation or promote immune evasion in the host (Nanbo et al., 2021). Current studies suggest that Siniperca chuatsi rhabdovirus (SCRV) can utilize the host miR-15b and miR-210 of teleost fish, whose expressions are pronouncedly enhanced during viral infection. SCRV participates separately in TBK1 and STING expression to manipulate IFN-I responses, promoting its replication and immune escape (Xu et al., 2018; Chang et al., 2020). In another research, Geddes et al. (2018) filter and determine the function of miR-576-3p in human hepatocarcinoma cell line HuH-7. With the expansion of the Oropouche virus, miR-576-3p decreases the expression of STING to restrict IFN-β related immune responses, which facilitates pathogenesis in the organism (Geddes et al., 2018). Feline herpesvirus 1 (FHV-1) also induces upregulation of cats miR-101 and miR-26a to stifle viral trespass in a cGAS-dependent way. These miRNAs target and repress the IFN-I-I negative regulator cytokine signaling 5 (SOCS5) to potentiate host immune response while preventing viral evasion (Zhang et al., 2019, 2020b). HCMV encoded miR-US33as-5p can bind IFNAR1 and dysregulate following ISGs expression. Accordingly, resistance to IFN-I induced viral elimination (Zhang Q. et al., 2021). Host DNA sensor Asp-Glu-Ala-Asp (DEAD)-box helicase 41 (DDX41) is capable of mediating STING-induced IRF3 activation (Briard et al., 2020). Besides, a recent study has identified that miR-H2-3p of HSV-1 downregulates the mRNA and protein level of DDX41 to affect IFN-β production, which promotes HSV-1 replication at the same time (Duan et al., 2019).

RNA regulatory mechanisms that participate in virus defense immunity are potential candidates for therapeutic targeting. Numerous non-coding RNAs remain unknown. Therefore, future studies need to pay more attention to these special RNAs.

## Severe Acute Respiratory Syndrome Coronavirus-2 Infection and Cyclic GMP-AMP Synthase-Stimulator of Interferon Genes

There are still numerous coronaviruses that have been uncovered and need to be analyzed (Hilgenfeld, 2014). In recent decades, the world has suffered from multiple coronaviruses, from SARS to the Middle East respiratory syndrome (MERS), and now, COVID-19. The world is expecting to find out effective antiviral therapeutics against this global pandemic. cGAS-STING is a pivotal antiviral pathway that has been recently proved by several studies of its involvement in SARS-CoV-2 infection (Berthelot and Liote, 2020; Berthelot et al., 2020; Liu et al., 2021). Here, we conclude many ongoing studies that focus on the cGAS-STING pathway as a therapeutic target to block the evasion of SARS-CoV-2.

Separate groups of researchers have reached an agreement that direct activation of STING can robustly block SARS-CoV-2 infection. Moreover, they found that the STING agonist, diABZI and diABZI-4, can effectively restrict SARS-CoV-2 replication (Chipurupalli et al., 2020; Zhu et al., 2021). Furthermore, Wu et al. (2021) expanded the function of a novel STING agonist, CDG^*SF*^, as an adjuvant for the SARS-CoV-2 vaccine. Compared with other coronaviral proteins, PLpro contributes to both virus replication and host cell signaling-cascade regulation, which is more suitable to be a target for antiviral drug design (Baez-Santos et al., 2015). Using protease activity-based and high-throughput screening methods, two valuable SCoV2-PLpro inhibitors, tanshinone IIA sulfonate sodium, and chloroxine, are selected and show their potential in clinical treatment for COVID-19 (Xu Y. et al., 2021). Ma et al. (2021) identify Jun9-72-2 and Jun9-75-4 as the representatives of several SCoV2-PLpro inhibitors, with higher affinity than previously reported inhibitor GRL0617. Another study has determined that combined administration of cGAMP and virus-like particles (VLPs) vaccine perform a worth noting effect in strengthening vaccine immunogenicity (Chauveau et al., 2021). These promising antiviral drug candidates shore up the tough struggle with the virus and encourage researchers to be more concerned about viral evasion mechanisms.

## Conclusion and Future Perspectives

The cGAS-STING pathway is evolutionarily conserved in mammalian species and has intriguing functions in other species (Cheng et al., 2020; Morehouse et al., 2020). Recent studies have revealed diverse regulation of the cGAS-STING pathway during virus infection and subsequent innate immune evasion of different viruses in distinct host species (Zheng, 2018; Zhu and Zheng, 2020; Guo Y. K. et al., 2021; Yu H. et al., 2021; Yu P. et al., 2021). Despite the conserved strategies we have concluded above, the emergence of more and more novel schemes shows the diversity and complicated network manipulation of cGAS-STING signal cascades during viral escaping, presenting a cat-rat race of survival between viruses and their hosts.

Viral immune escape factors craftily control cGAS-STING signal transduction from beginning to end. Intriguingly, at the first step of infection, human papillomaviruses (HPVs) have evolved a special vesicular trafficking method, which can translocate viral genome (vDNA) into host intranuclear environment without being detected by surveillance of abnormal DNA ligands, therefore bypassing the cGAS-STING pathway (Uhlorn et al., 2020). Then, the second messenger, cGAMP, is produced after detecting viral DNA and acts as the immunostimulator of the cGAS-STING pathway. Poxvirus immune nucleases (poxins) from mammalian and insecticidal poxvirus have been recently defined for their participation in cGAMP degradation. They have also abolished downstream STING signal cascades reaction (Eaglesham et al., 2019, 2020). Several processes also take part in regulating key component functions of the cGAS-STING pathway. Marek’s disease virus (MDV) major oncoprotein Meq hampers the combination of IFN regulatory factor 7 (IRF7) and TBK1 with STING, which facilitates MDV-induced lymphomagenesis in avians (Li et al., 2019). Another finding that was previously reported is that capsid protein of MDV, VP23, also participates in the cGAS-STING blockade by impeding TBK1 phosphorylation of IRF7 (Gao et al., 2019). The DP96R gene of the African swine fever virus (ASFV) suppresses TBK1 phosphorylation and inhibits IKKβ, contributing to the evasion of ASFV from immune clearance (Wang et al., 2018). A recent study uncovers that the interaction of STING with sulfated glycosaminoglycans (sGAGs) is essential to exercise the STING function. Decreased expression of Slc35b2 hampers the sulfate process of GAGs. Thus, the STING polymerization is impeded, blunting the immune responses to vaccinia virus infection (Fang R. et al., 2021). Presenting these evasion tactics of the virus provides insight into targeting novel antiviral countermeasures.

Apart from adjusting signal delivery by key proteins, several strategies regulate the intensity of antiviral response at the mRNA level. Li et al. (2021a) suggest that the RNA-binding protein LUC7L2 participates in the negative feedback of virus-induced immunity by interaction with STING precursor mRNA, which represses STING expression. IFN-I signaling activation also induces proteasome degradation of WT1-associated protein (WTAP) to decrease m6A modifications of IRF3 and IFNAR1 mRNAs, which negatively regulates antiviral responses (Ge et al., 2021). Moreover, recent findings also reveal that people of STING haplotype are more sensitive to dengue virus (DENV) protease than homozygote genotype, whose STING is risky to be cleaved during viral infection. This research discusses how the diverse STING genetic background affects DENV pathogenesis and provides another orientation for future precision medicine development (Su et al., 2020).

Viruses are the mainspring of infectious diseases, several carcinogenic processes, and have caused immeasurable public health for years. Understanding how viruses adjust the innate immune system affords probabilities to cure virus-related diseases and prevent viral infection. However, simply knowing the principles and patterns is merely a beginning. Antiviral drug development is an urgent issue to humanity to diminish the impact of virus disturbance and prevent the evasion of viruses. Acting as the key checkpoints in maintaining the interferon homeostasis *in vivo*, several valuable targets in cGAS-STING signal transduction offer inspiration for antiviral drugs invention.

Recently, multiple anticancer drugs have been repurposed of their capabilities in antiviral treatments (Aldea et al., 2021; Xu Y. et al., 2021). For instance, β-arrestin 2 is a regulator of G protein-coupled receptor (GPCR) signaling pathways, promoting cGAMP production to regulate the cGAS-STING axis by targeting cGAS positively. During the viral invasion, β-arrestin 2 is degraded by the ubiquitin-proteasome system, which causes the decrease of IFN-β level in host cells and viruses evasion. Apart from its known efficacy in curing heart disease, Carvedilol is re-screened as the blocker of virus-induced β-arrestin 2 degradations to rescue the diminished antiviral immune response, which provides a novel candidate for antiviral drug research and development (Zhang et al., 2020d).

Notwithstanding all these mechanisms about virus escape we have discussed above, there are still innumerable details that need further exploration. The appearance of drug-resistant variants and novel viruses with high pathogenicity are bound to immeasurable economic and public health damages. There still is a long way to go with this tug of war between humans and viruses.

## Author Contributions

TH drafted the manuscript, and prepared figures with BioRender.com. YY, MP, QW, and YC revised the manuscript. CW edited and reviewed the final version of this manuscript. All authors contributed to the article and approved the submitted version.

## Conflict of Interest

The authors declare that the research was conducted in the absence of any commercial or financial relationships that could be construed as a potential conflict of interest.

## Publisher’s Note

All claims expressed in this article are solely those of the authors and do not necessarily represent those of their affiliated organizations, or those of the publisher, the editors and the reviewers. Any product that may be evaluated in this article, or claim that may be made by its manufacturer, is not guaranteed or endorsed by the publisher.
